# Coffee bean sign: Its meaning and importance

**DOI:** 10.1002/ccr3.3064

**Published:** 2020-06-26

**Authors:** Eliza Stavride, Charalampos Plakias

**Affiliations:** ^1^ Department of Radiology Papageorgiou General Hospital Thessaloniki Greece

**Keywords:** coffee bean sign, dilated bowel, sigmoid volvulus, vovlulated sigmoid colon

## Abstract

The presence of the coffee bean sign is pathognomonic of sigmoid volvulus.

## Graphical abstract

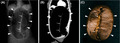

The coffee bean sign is a radiological sign used to describe the twisting of the sigmoid colon about its mesenteric axis, mimicking the picture of a coffee bean. Physicians should be aware of this sign and when it is present, they should consider emergency intervention, since sigmoid volvulus is a potentially life‐threatening condition.

A 57‐year‐old, disabled man presented to our emergency department with symptoms of progressive abdominal pain and acute constipation. Physical examination revealed abdominal distension, generalized tenderness, and hypoactive bowel sounds. Plain radiograph in left‐sided position revealed dilated colon loops with air‐fluid levels. However, the scout view image of the computed tomography scan depicted an impressive picture of a “coffee bean” sign (Figure 1A). The examination revealed a twisted loop of sigmoid colon with marked distension (Figure 1B). Sigmoid volvulus was diagnosed rapidly. Subsequent colonoscopy failed to decompress the bowel (Figure 2), leading to the immediate decision to take the patient to the theater. Upon laparotomy, the sigmoid colon was found to be necrotic due to volvulated mesentery, leading to resection.

**FIGURE 1:**
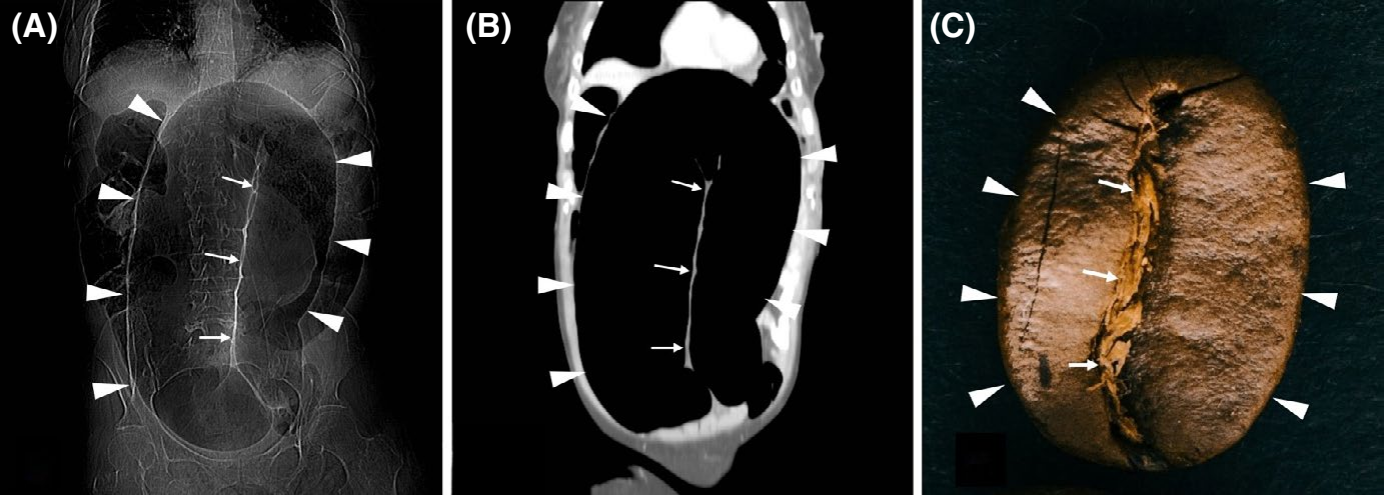
A computed tomography scout view (A) and a coronal image (B) demonstrating the picture of a coffee bean (C). The two parts of the coffee bean represent the gas‐filled segments of the dilated bowel (arrowheads), whereas the central cleft of the coffee bean represents the double thickness of opposed bowel walls (arrows)

**FIGURE 2:**
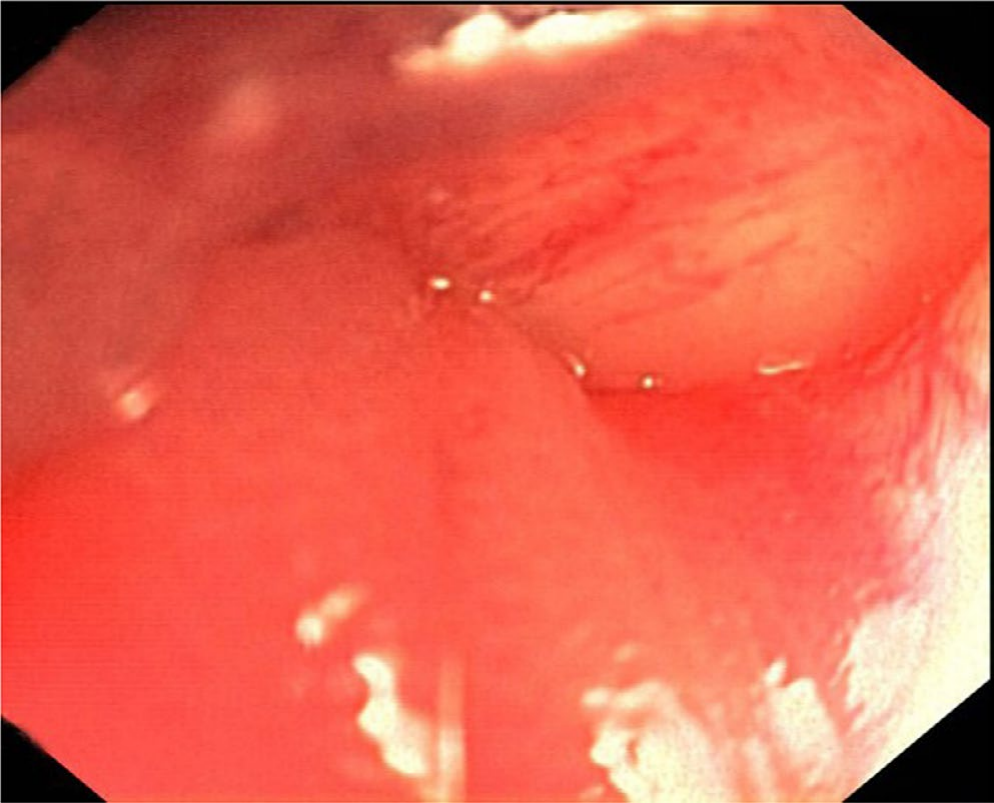
Endoscopic picture of the volvulated sigmoid colon

The coffee bean sign is a radiological sign used to describe the twisting of the sigmoid colon about its mesenteric axis, mimicking the picture of a coffee bean (Figure 1C). The two side parts of the bean represent the gas‐filled segments of the dilated bowel creating an inverted U‐shape, whereas the central cleft of the bean represents the double thickness of opposed bowel walls.^1^ The presence of air within the bowel wall is a sign known as parietal pneumatosis and it is suggestive of bowel ischemia, whereas the presence of extraluminal air in the peritoneal cavity suggests bowel perforation. Chronic constipation, anatomic variations, neurologic diseases, and megacolon are the main risk factors of sigmoid volvulus in adults. Depiction of this sign should alert physicians to consider emergency intervention.^2^

## CONFLICT OF INTEREST

None declared.

## AUTHORS' CONTRIBUTION

ES: reviewed the literature, wrote the manuscript, and edited the images. CP: contributed to patient care, made a contribution to drafting, and reviewed the manuscript.
